# Comparison of NIH 3T3 Cellular Adhesion on Fibrous Scaffolds Constructed from Natural and Synthetic Polymers

**DOI:** 10.3390/biomimetics8010099

**Published:** 2023-03-01

**Authors:** Katarina McGarry, Eelya Sefat, Taylor C. Suh, Kiran M. Ali, Jessica M. Gluck

**Affiliations:** Department of Textile Engineering, Chemistry and Science, Wilson College of Textiles, North Carolina State University, Raleigh, NC 27606, USA

**Keywords:** collagen, PLA, tissue engineering, cell adhesion, cell proliferation, biomimetic materials

## Abstract

Polymer scaffolds are increasingly ubiquitous in the field of tissue engineering in improving the repair and regeneration of damaged tissue. Natural polymers exhibit better cellular adhesion and proliferation than biodegradable synthetics but exhibit inferior mechanical properties, among other disadvantages. Synthetic polymers are highly tunable but lack key binding motifs that are present in natural polymers. Using collagen and poly(lactic acid) (PLA) as models for natural and synthetic polymers, respectively, an evaluation of the cellular response of embryonic mouse fibroblasts (NIH 3T3 line) to the different polymer types was conducted. The samples were analyzed using LIVE/DEAD™, alamarBlue™, and phalloidin staining to compare cell proliferation on, interaction with, and adhesion to the scaffolds. The results indicated that NIH3T3 cells prefer collagen-based scaffolds. PLA samples had adhesion at the initial seeding but failed to sustain long-term adhesion, indicating an unsuitable microenvironment. Structural differences between collagen and PLA are responsible for this difference. Incorporating cellular binding mechanisms (i.e., peptide motifs) utilized by natural polymers into biodegradable synthetics offers a promising direction for biomaterials to become biomimetic by combining the advantages of synthetic and natural polymers while minimizing their disadvantages.

## 1. Introduction

The field of tissue engineering aims to repair and/or regenerate tissues damaged from age, disease, trauma, or congenital defects [1]. Tissue engineering consists of three components: cells, scaffolds, and growth signals. The rapidly developing field holds the potential to help many individuals who have a poor quality of life due to these injuries and illnesses. While a loftier goal of tissue engineering may be to replace organs, the current literature focuses heavily on the use of polymeric materials for drug delivery and the ability of the materials to improve healing. As research into biomaterials continues to grow, it is important to understand the exact attributes of materials that allow them to meet the unique needs of clinical challenges.

Natural polymers are commonly used for scaffolds in tissue engineering. These polymers are found in nature and are typically extracted from plants and animals [1]. Due to their origin, these polymers tend to exhibit desired interactions with cells when implanted in the body [2] and thus tend to induce superior cell adhesion, migration, and proliferation when used as tissue engineering scaffolds. Across industries, natural polymers are implemented in different methods. In traditional textiles, natural polymers such as cellulose are made into viscose rayon or cellulose acetate fibers. In other material industries, natural rubber from tree latex is often vulcanized and used for conveyor belts [3]. In biomedical research, there is ample evidence that incorporating natural polymers into biomaterials improves the bioactivity of the engineered material [4]. Since the creation of the term of tissue engineering in the 1980s by Langer and Vacanti, researchers have been using natural polymers for tissue engineering scaffolds [5]. However, many questions persist surrounding the exact mechanisms that allow for such bioactivity in a case-by-case manner.

The significance of understanding natural polymers’ interaction with biological systems comes from their tendency to elicit immune responses and the increased use of biodegradable synthetic polymers to counter this issue [6]. The fourth generation of biomaterials has come to focus significantly on biomimetic materials while having an increased use of synthetic polymers [7]. This polymer group comprises man-made polymers that are biocompatible and tend to be degradable by simple hydrolysis [8]. This makes them very useful in tissue engineering because they can still be tailored for specific applications such as wound closure devices such as sutures or orthopedic fixation devices such as pins, rods, or screws [9].

This study aims to compare how successfully cells adhere and proliferate on scaffolds produced by natural polymers compared to biodegradable synthetic ones. Specifically, we utilize fibrous scaffolds because these polymeric materials are widely popular for tissue engineering [10]. Fibrous approaches have been shown to be better at mimicking the extracellular matrix (ECM) and providing more surface area for cellular attachment [10]. We seek to gain any insight into the differences in natural polymers that may provide enhanced bioactivity in relation to the overall process of developing biomaterials. Understanding these mechanisms will help improve our understanding of what makes tissue engineering scaffolds biomimetic and will thus allow us to harness the “best of both worlds” from both synthetic and natural polymers to create more effective and functional fibrous scaffolds for drug delivery and clinical use.

To conduct this comparison, a model for each polymer class had to be selected. In the case of natural polymers, collagen will be used as a model because it is the most abundant protein in the human body [11]. Additionally, collagen exhibits excellent biocompatibility, low antigenicity, and appropriate hemostatic properties for various tissue engineering applications [12]. While these attributes make collagen attractive for scaffolds, the polymer is not without its disadvantages. These include mechanical weakness [13] and variability in properties depending on the collagen source [12]. Collagen also degrades quickly. Depending on the end-use of the scaffold, this rapid degradation can be considered an advantage or disadvantage. Typically, the degradation rate of a scaffold should match the de novo tissue formation at its site of use or implantation [12,14]. Because collagen exhibits advantages and disadvantages characteristic of most natural polymers, we will use it to represent these natural polymers in our comparative study. With the end goal of tissue engineering being a replica of tissue systems in human physiology, collagen offers an excellent model for comparison.

The model that will be used for biodegradable synthetic polymers is polylactic acid (PLA). This is highly biocompatible due to the material’s properties regarding degrading into lactic acid, carbon dioxide, and water [15]. These compounds are common in the human body, allowing the material not to elicit an inflammatory response initially when implanted. We selected PLA to represent synthetic polymers in this study because, much like how collagen exhibits many key advantages and disadvantages of natural polymers, so does PLA for synthetic ones. These advantages include the aforementioned high biocompatibility, as well as the high biodegradability, excellent mechanical and barrier properties, relatively low cost, and high tunability, which can be controlled during production or post-processing [16]. This allows for the more precise and controllable mimicry of the extracellular matrix. However, PLA and other synthetic polymers usually exhibit inferior cell adhesion, migration, and proliferation compared to collagen and other natural polymers. Additionally, PLA can induce inflammatory responses to its acidic byproducts during degradation [17].

## 2. Materials and Methods

All chemicals and reagents were purchased from ThermoFisher (Waltham, MA, USA) unless otherwise noted.

### 2.1. Collagen and PLA Fibrous Scaffold Creation

Collagen Scaffold Fabrication: Bovine collagen fibers (donation of Kaneka Corporation) were processed through a card chute system (Reiter Card C4), followed by two rounds of drawing (Reiter RSB851) until the fibers were anisotropically aligned. They were then passed through the roving machine (Reiter Fly F4/1) and then a ring spinning machine (Reiter G5/2), where yarns of 265 denier (yarn thickness unit) were produced [18,19]. For this experiment, 12-inch pieces were cut and bundled up to make a tangled network of yarns. The samples were sterilized by soaking these yarns in a 70% ethanol solution and rinsing them with Dulbecco’s Phosphate-Buffered Saline (PBS) (DPBS, Cytiva). Each sample of yarn bundles was then placed into the wells of a 24-well plate. These yarn bundles are henceforth referred to as “collagen scaffolds”.

PLA Scaffold Fabrication: PLA fibers were provided by Xinxiang Sunshine Textiles Co., Ltd., Xinxiang, China, in yarn form (150 denier PLA yarn). The PLA yarn samples were spun into a yarn-like structure and processed with the same methods previously described for the collagen scaffold fabrication [19]. Twelve-inch pieces were cut and tangled into weblike bundles before being sterilized with 70% ethanol and washed with PBS. These weblike bundles are henceforth referred to as “PLA scaffolds”.

### 2.2. Scanning Electron Microscopy

A scanning electron microscope (SEM) examines visual growth using secondary and backscattering scans. This provides an image of the topography and a deeper view of the surface to visually assess cellular attachment, proliferation, and migration into the scaffold while also characterizing the scaffold itself. Images were taken on days 1 and 7, following sample fixation, which was conducted by washing the seeded samples with PBS and then fixing them by submerging them in an SEM buffer comprising 4% paraformaldehyde for approximately 20 min. The samples were then washed with PBS two to three times and then a 0.1 M sodium cacodylate buffer, pH 7.2, supplemented with 5% sucrose for 15 min. They were then dehydrated with a progressively increasing amount of ethanol: 35%, 50%, 70%, 80%, 95%, and 100% diluted to the appropriate concentration in water; 35–95% are in intervals of 10 min, and the first 100% is in intervals of 3 by 10 min of washing, followed by a 40 min wash. After dehydration, the samples were soaked in a solution comprising a 1:1 ratio of 100% EtOH and hexamethyldisilane (HMDS) for 20 min and then in pure HDMS for 20 min. Once the samples were dried, they were mounted on imaging stubs with carbon tape.

### 2.3. NIH 3T3 Cells

The cells used for this study were NIH 3T3 fibroblasts. These embryonic murine fibroblasts are representative of a type of common cell found in connective tissues and an immortalized cell line which would allow for a generic comparison. The cells were cultured under static conditions and maintained in media consisting of Dulbecco’s modified eagle medium, 10% fetal bovine serum, and 1% penicillin-streptomycin. The media were changed every 72 h [20]. The cells were passaged regularly using Trypsin—0.25% EDTA (Gibco, ThermoFisher, Waltham, MA, USA) upon reaching approximately 80% confluence. These cells were thawed at passage 12, plated onto plasticware coated with 0.1% gelatin, and then incubated at 37 °C and 5% CO_2_. Once at the appropriate confluency for providing the correct seeding density was reached, the cells were counted and seeded onto scaffolds at a density of 100,000 cells/cm^2^, with control wells (a well that contains only cells and no scaffold) seeded with 50,000 cells/cm^2^ to prevent overgrowth. A higher number of cells were plated in the wells containing scaffolds to account for those cells that would adhere to the 3D volume of the scaffolds rather than the tissue culture plastic. To ensure maximum cell adhesion, the total number of cells were suspended in 20 μL of media, added to the collagen or PLA samples, and incubated for 20 min before a total volume of 1 mL of media was added to each well.

### 2.4. Cell Viability Testing

A LIVE/DEAD™ Cell Imaging Kit (488/570) (R37601, ThermoFisher, Waltham, MA, USA) analyzes the cell viability by measuring the cytotoxicity, intracellular esterase activity, and plasma membrane integrity. Calcein dye stains live cells bright green and ethidium-homodimer 2 stains dead cells red. The live cells turn green because of the enzymatic conversion of the non-fluorescent calcein to fluorescent calcein, which indicates intracellular esterase activity [20]. The dead cells turn red because of binding to DNA when the dye gets through the damaged membrane. To test the cell viability, seeded samples were tested on days 1, 3, 5, and 7 post-seeding. After incubation, the samples were imaged with an EVOS FL Auto 2 (ThermoFisher) fluorescent microscope.

On the same days as the LIVE/DEAD™ assay, an alamarBlue™ assay was conducted to quantify cellular proliferation. The reagents were added to the complete media and incubated for 1–4 h. The alamarBlue™ Cell Viability Reagent is a non-toxic resazurin dye that enters the cell membranes. It is initially blue and non-fluorescent. However, once it enters a living cell, it is metabolized to resorufin, which is red and fluorescent. Therefore, cellular proliferation can be detected through the absorbance of 570 and 600 nm or the fluorescence excitation of 530–560 nm and the emission of 590 nm of the cells. The plates were incubated for 90 min and then read using a microplate reader (Synergy HT, BioTek, Santa Clara, CA, USA) set to 540/25 λ excitation and 590/35 λ emission and maintained at 37 °C.

### 2.5. Immunofluorescent Staining

Phalloidin immunofluorescence stain was used to elucidate the integrity and morphology of the cells’ cytoskeletons and to corroborate the cellular viability results from the LIVE/DEAD, alamarBlue, and SEM image results. Phalloidin dye has a high affinity for filamentous F-actin within the cytoskeleton. Thus, the dye stains the cytoskeleton and emits green fluorescence with excitation at 495 nm and emission at 518 nm. The samples were fixed on days 3 and 7 by a PBS wash, followed by submergence in 4% paraformaldehyde for approximately 20 min. Next, the samples were permeabilized with 0.20% TritonX-100 for 30 min and washed in PBS + 0.1% Tween-20 for 5 min. This step was repeated three times. The samples were then incubated in a blocking buffer comprising 2% Bovine Serum Albumin (BSA) and 2% goat serum in PBS + 0.1% Tween-20. Two drops per mL of Invitrogen™ ActinGreen™ 488 ReadyProbes™ Reagent containing the phalloidin stain were added to the blocking buffer. A total of 250 μL of the solution was added to each sample, and the samples were incubated for an hour. The samples were then washed with PBS and stained with Hoechst for 5 min at a concentration of 30 mL PBS and 30 μL Hoechst in order to allow for the imaging of the cell nuclei. Three washes of PBS were carried out, and two drops of Invitrogen ProLong Gold antifade solution (ThermoFisher) were added to each sample with 200 μL of PBS. The samples were then imaged using the FL Auto 2 fluorescent microscope (ThermoFisher).

## 3. Results

### 3.1. Scaffold Characterization

**Figure 1:**
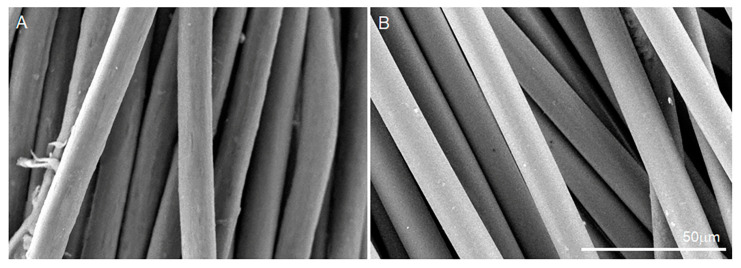
Representative SEM images of (**A**) collagen and (**B**) PLA scaffolds at 1000× magnification. Scale bars = 50 µm.

SEM utilizes electrons to scan the surface of the matrices. The basic morphology of the yarns created from the collagen and PLA fibers is observed in Figure 1. Representative SEM images of the natural collagen and synthetic PLA scaffolds (Figure 1) elucidate their morphology. Furthermore, we quantified the fiber diameters for each sample and found mean fiber diameters of 12.562 ± 1.352 μm for collagen scaffolds and 12.595 ± 0.753 μm for PLA scaffolds. No statistical significance was observed between the fiber diameters of the collagen and PLA scaffolds. Both collagen and PLA had similar-sized fiber diameters, but the collagen samples contained a greater variation in the fiber diameter.

### 3.2. Cell Attachment Results

**Figure 2:**
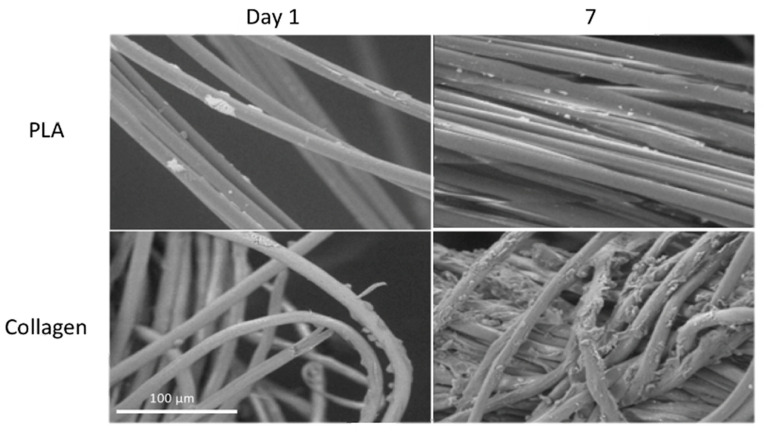
Representative SEM images of cells maintained on collagen and PLA scaffolds at days 1 and 7 with 500× magnification. Cells adhered along the length of the collagen yarns at day 7, compared to the limited cell adhesion on PLA yarns at the same timepoint. Scale bars = 100 µm.

To assess the cell attachment, SEM images were captured following the fixation of collagen and PLA scaffolds on days 1 and 7 post-seeding with NIH 3T3 fibroblasts (Figure 2). The surfaces of the collagen and PLA scaffolds seeded with NIH 3T3 cells are shown in Figure 2. The images of the collagen scaffold display an increase in cell coverage from day 1 to day 7. This indicates that the cells adhered to and proliferated on the scaffold. These images also elucidate some of the morphology expressed within the scaffolds’ microenvironments, namely, they show the fibers within the scaffolds. This texture will influence the surface roughness properties because, at the site of intersections between fibers, the scaffolds have imperfections, dips, and valleys. These create pores that are desirable for cellular attachment and proliferation because they increase the scaffold’s surface area. 

Compared to the collagen scaffold, the PLA scaffold does not show as many cells attached to the scaffold. From day 1 to day 7, the number of cells decreases according to the visual assessment, whereas, on day 7, the cells appear fragmented rather than healthy and whole. Additionally, this yarn-like structure appears to be smoother than that of the collagen fibers. This is evident because no visual texture is visible on the collagen scaffold itself. The cells adhered along the length of the fibers within the collagen scaffold and proliferated throughout the scaffold by day 7.

### 3.3. Cell Viability Results

**Figure 3:**
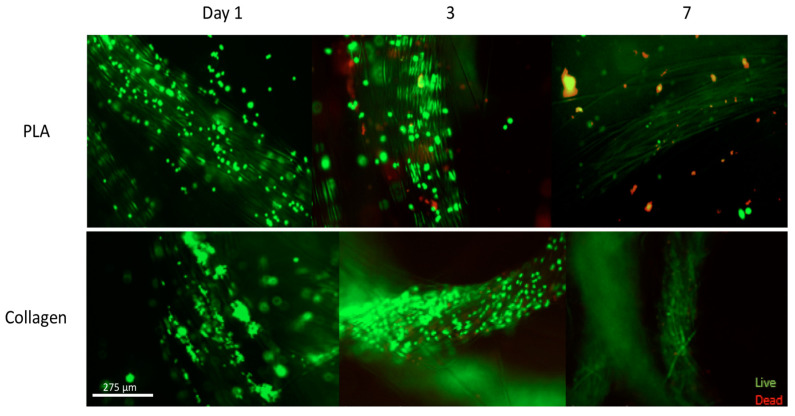
Representative Live/Dead Images of Collagen and PLA samples. Images were acquired at days 1, 3, and 7 for both samples. Live cells appear green and dead cells appear red. Scale bar = 275 μm.

To test for basic biocompatibility, a LIVE/DEAD™ assay was conducted. Between day 1 and day 3, the cell quantity on the collagen scaffolds increased, indicating proliferation (Figure 3). The image shown is representative; the cells proliferated similarly across the entirety of the collagen scaffold, covering a majority of its surface, as confirmed by visual assessment. Visual assessment also confirmed the adhesion of the cells to the collagen scaffolds, indicating that the cells found the microenvironment provided by the scaffolds favorable. Very few dead (stained red) cells are visible, most of which did not adhere to the collagen scaffolds. The fact that the dead cells were the ones that failed to adhere indicates that the attachment to the collagen scaffolds promoted cell survival. However, on day 7, the number of living cells decreased. This is likely due to a combination of two reasons: first, living cells migrated into the inner structure of the collagen scaffolds, where they are no longer visible due to the scaffolds’ opacity. Second, cellular proliferation typically slows or halts when confluency is reached, primarily due to the limited surface area for new cellular attachment (referred to as contact inhibition).

The cells seeded onto the synthetic PLA scaffolds showed the highest quantity of living cells, as well as the highest ratio of living cells to dead cells, on day 1. On this day, the cells were shown to have adhered quickly and efficiently to the PLA scaffolds, with virtually no dead cells. This rapid adhesion indicates that PLA provides a sufficiently biocompatible environment for NIH 3T3 cells to anchor onto. However, by day 3 and day 7, there was a notable decrease in the cellular quantity, as well as a notable decrease in the living-to-dead-cell ratio. Therefore, we can conclude that while the synthetic PLA scaffold may offer sufficient biocompatibility for facilitating cellular attachment, it may not have a sustainable long-term microenvironment that promotes cell migration and proliferation, in contrast with the natural collagen scaffold.

**Figure 4:**
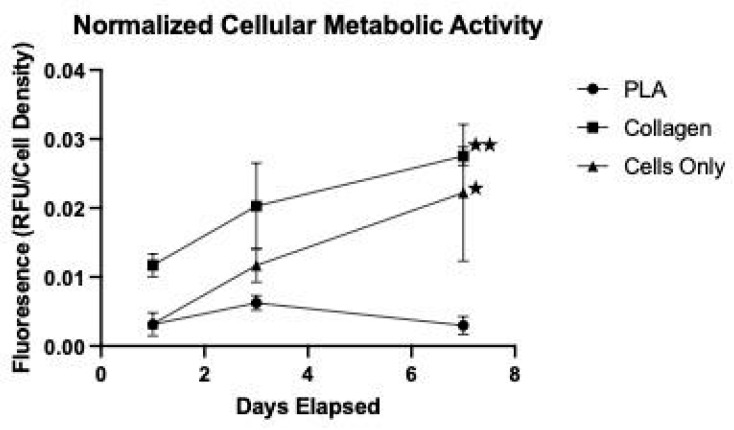
Cellular Proliferation by the Metabolic assay, as measured in arbitrary fluorescence units (RFU) indicated by the alamarBlue™ assay. The RFU values were normalized in relation to the cell density plated. The error bars represent standard deviations. ★ indicates a statistically significant difference between the Cells-only and PLA samples at *p* < 0.05. ★★ indicates a statistically significant difference between the Collagen and Cells-only samples at *p* < 0.05 (*n* = 3 for all samples).

The data from the alamarBlue™ plate readings were averaged and compiled to form Figure 4. These graphs illustrate the trends of metabolic activity in the cells over the 7-day testing period. A fluorescent plate reader was used to quantify the cellular metabolic activity and thus proliferation based on fluorescence, as measured in arbitrary units.

The NIH 3T3 cells on the natural collagen scaffolds and in the control well exhibited a positive trend over the testing period. The metabolic activity of the cells seeded onto the natural collagen scaffolds exhibited a steep slope with a fast-rising fluorescence value, indicating rapid proliferation, which is consistent with the results from the LIVE/DEAD™ assay (Figure 3). Conversely, the metabolic activity of the cells seeded on the synthetic PLA scaffolds exhibited a decreasing trend. It rises slightly in the first three days, only to drop to around the starting value. Again, this is consistent with the LIVE/DEAD™ assay results for the cells on the PLA scaffolds and indicates that while the synthetic PLA scaffolds offer a sufficiently biocompatible microenvironment for initial cell attachment, they fail to support long-term cellular proliferation. The cells-only series is the cells-only well in all plates and shows a positive trend, indicating that the cells did grow over the trial in the absence of any scaffold.

### 3.4. Immunofluorescent Staining Results

**Figure 5:**
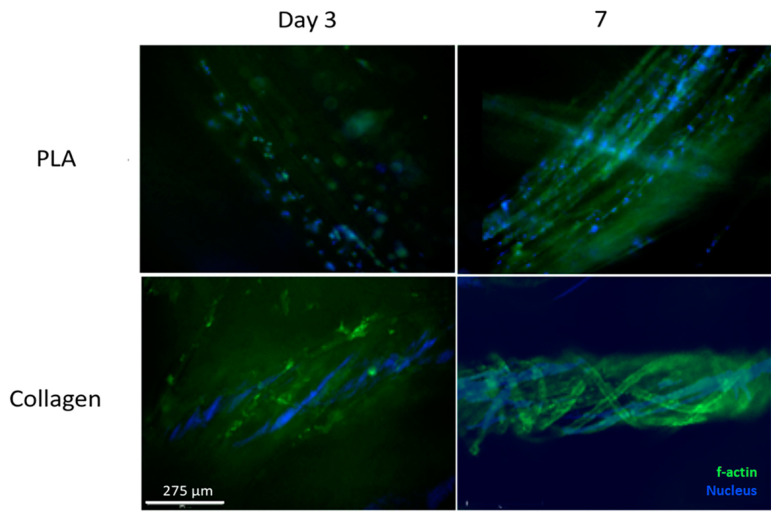
Representative Phalloidin-stained Collagen and PLA Samples. Images were acquired on days 3 and 7. Green indicates f-actin in the cytoskeleton of the NIH 3T3 cells (phalloidin stain), and blue indicates cellular nuclei. Scale bar = 275 μm.

Phalloidin staining aims to examine the cytoskeleton of cells. The high-affinity filamentous F-actin, a protein found in the cytoskeleton, is illuminated green, while the nuclei appear blue due to Hoechst counterstaining. Figure 5 illustrates the green F-actin and blue nuclei along the collagen and PLA scaffolds. The images indicated the presence of cells on the scaffolds, with a significant number of nuclei in the scaffolds, indicating cell attachment.

## 4. Discussion

Consistent with previous studies and theory, the results indicate that the cells preferred natural collagen scaffolds to synthetic PLA scaffolds. The LIVE/DEAD™ assay indicated an increase in the cell viability of the collagen samples from day 1 to day 7 (Figure 3). A visual assessment of the images suggests that the cells dispersed throughout the collagen scaffolds, migrating throughout the fibers. This suggests that the cells found the microenvironment provided by the collagen scaffolds more favorable. In addition, very few dead cells were present, indicating that the collagen scaffolds introduce little to no cytotoxicity. Notably, the dead cells are not attached to the scaffolds, indicating that those that did die likely failed to attach to the scaffold and thus died not due to a lack of scaffold biocompatibility but rather due to a lack of structural support. This is consistent with previous studies which indicate that collagen is biocompatible with the NIH3T3 cells and aids in cell adhesion and proliferation [18] and the use of collagen scaffolds in a wide range of applications, from nanostructured mats electrospun alongside glycosaminoglycans for nerve tissue regeneration [21] to promoting osteogenesis and differentiation for bone tissue engineering [22].

The images for the PLA scaffolds and NIH 3T3 cells seeded onto them had a different trend. As the test progressed, the density of cells appeared to decrease over time. The cells adhered to the PLA scaffolds during the first day, but the cells detached and died as time progressed. PLA did not support the conditions necessary for maintaining long-term cell adhesion. Phalloidin staining images also supported the trends in adhesion. On day 7, the cells appeared to cover the collagen scaffolds and multiply over the testing period, while in contrast, the cell density decreased in the PLA samples. This suggests that while the synthetic scaffolds did not present cytotoxicity to the cells, they failed to promote the successful attachment and migration of the cells into the scaffolds and thus did not provide a suitable microenvironment for cellular proliferation.

The difference in cellular adhesion between the collagen and PLA samples suggests the existence of a structural difference between the polymer samples that cause different cellular interactions. Collagen is a protein that consists of amino acids, while PLA consists of lactic acid. A 2018 study by Kang and colleagues on modified peptides suggested that incorporating more Arginine–Glycine–Aspartate (RGD) amino acid motifs into films provided greater cellular adhesion [23]. This was built upon previous work that indicated that the RGD sequence is essential to the cellular recognition of fibronectin and has been found to be the case in multiple natural polymers such as laminin, vitronectin, and fibrin [24]. Collagen type I contains one or more of these sequences, so it is possible that the RGD sequence is responsible for its greater cellular adhesion [24].

It is important to note that other factors are also important to the cellular adhesion to a fibrous biomaterial. Material properties such as the fiber diameter, fiber arrangement, cross-sectional shape, and surface hydrophobicity have all been shown to affect cellular adhesion to materials [25]. Fiber diameters larger than those of cells allow cells to extend along fibers, while diameters smaller than those of cells allow them to wrap around fibers [25]. Looking at Figure 2, the fiber diameters between the collagen (12.562 ± 1.352 μm) and PLA (12.595 ± 0.753 μm) samples were relatively similar, with no significant difference. Based on that similarity, it is less likely that general material properties were responsible for the significant difference in adhesion between the samples. In addition, the SEM images in Figure 2 also indicate that cells adhere along the length of the fiber, regardless of the yarn bundling. It is safer to assume that the most dominant reason for the adhesion differences is structural differences in the polymers used.

Beyond adhesion, the alamarBlue™ data indicated that cellular metabolic activity was also stronger with the collagen samples. This is evident with the much larger average fluorescence values seen in the collagen samples. This means that more cells were proliferating and content in their environment. On the other hand, the trend in the PLA alamarBlue™ data indicated that the cells proliferating on the scaffold decreased over time. In fact, the metabolic activity on the PLA sample was worse than that in the control well. This difference is likely due to the PLA samples having fewer live cells due to less adhesion.

The current understanding of molecular biology indicates that cellular metabolic activity is heavily influenced by cellular adhesion to a bigger extracellular matrix (ECM) [26]. For cells such as epithelial, endothelial, and muscle cells, previous studies indicate that cells that fail to adhere to the ECM or lose contact with the matrix undergo apoptosis [26]. With that reference, the difference in metabolic activity between the cells on the collagen and the PLA samples was likely rooted in their structural ability to encourage cellular adhesion and the formation of an ECM. This amplifies the importance of the ability of biomaterials to mimic cellular binding mechanisms for applications such as organ replacement or clinical challenges that are wound-healing in nature.

There are attempts in biomaterials that utilize the idea of tuning synthetic scaffolds to be more biomimetic through the use of natural polymers. For keratoprosthesis challenges, di-amino-PEG that contains the RGD peptide has been grafted onto PMMA surfaces and has restored cellular adhesion, with enhanced attachment compared to untreated PMMA [27]. The same motif was utilized as a graft onto the surface of Poly(Carbonate-Urea)urethane and improved hepatocyte adhesion [28]. Besides the most common RGD motif, the laminin motif IKVAV has been utilized in hydrogels to promote neurogenesis [29]. An excellent review by Li and colleagues touches on incorporating bioactive ingredients in biomaterials for spinal regeneration [30].

Despite the evidence for incorporating cellular binding mechanisms into tissue engineering, there are some limitations. Incorporating complete proteins increases the opportunity for immunogenic responses and infection. This is due to their susceptibility to proteolytic degradation and the presence of inflammation and infection in vivo, accelerating protein degradation [31]. Most current attempts utilize presenting motifs that cells recognize in the form of immobilized peptides [31]. Thus, it is imperative to probe the role of specific motifs in signaling cascades that result in increased cellular attachment and proliferation. This understanding will lead us to more efficient and biomimetic scaffolds for tissue engineering, which can ultimately be used for various applications, including wound healing, the repair and replacement of damaged tissues, and microfluidic models.

Outside of the binding considerations, some additional challenges need to be considered when designing more biomimetic scaffolds. While textile technology approaches tend to offer the advantages of mimicking the ECM and the anisotropic and strain-stiffening tendencies seen across human physiology, there are struggles among preparation techniques [32]. Specifically, many reviews have indicated that electrospinning is currently the most dominant scaffold preparation in the modern tissue engineering literature but has major challenges with industrial replication due to Rayleigh, axisymmetric, and whipping instability [32,33]. Attempts at 3-D biomimetic scaffolds involve different woven, kitted, and braided patterns [25]. An interesting future direction for biomimetic materials and tissue engineering is utilizing a commercially viable, nanofibrous fabrication technique with bioactive ingredients such as peptide motifs.

## 5. Conclusions

Mouse fibroblasts presented with a higher bioactivity on collagen yarn fibers over PLA fibers. This is consistent with previous literature on the biocompatibility of fibrous collagen scaffolds. The results suggest structural differences between naturally derived polymers and biodegradable synthetic polymers that allow for better cell adhesion, proliferation, and metabolic activity. Future biomaterials attempts should incorporate peptide motifs that improve bioactivity into biodegradable synthetic-based materials.

## Data Availability

Not applicable.
